# Editorial: Insights into the caregiver perspective: involvement, well-being, and interventions

**DOI:** 10.3389/fpsyt.2023.1203350

**Published:** 2023-05-11

**Authors:** Shulamit Ramon

**Affiliations:** Department of Allied Health, Midwifery and Social Work, University of Hertfordshire, Hatfield, United Kingdom

**Keywords:** caregiving, rewarding caregiving, demanding caregiving, physical illness, mental illness

The range, duration, and intensity of informal caregiving across different illnesses and disabilities have increased in the 21st century due to the increase in longevity and de-institutionalization in many countries. De-institutionalization has led to increase in the demand of and ability to provide homecare in the community by informal caregivers.

Caregiving is demanding, and hence can be stressful in terms of time, effort, emotional and social impact, as well as financial requirements, depending on the nature of the illness or disability, the relationships between the person supported and the caregiver, and the role played by available health and social care services. Yet, research evidence has demonstrated that caregiving can be also rewarding, as a different type of bonding is enabled than was the case before caregiving became a necessity.

The thirteen articles published in this issue on the theme of caregiving make for an original in-depth contribution to existing research on this sensitive and important issue.

The articles cover a range of countries (Canada, China, India, Italy, Malaysia, Singapore, Spain, UK, and US), and a wide range of physical and mental ill health issues which caregivers are responding to, leading to differentiated impact on them too.

The issues looked at include caregiving to children and adults, focusing on:

- Borderline Personality Disorder,
- Chronic illnesses,
- COVID-19,
- Down syndrome,
- First Episode of Psychosis,
- Lung cancer,
- Recovery from mental illness,
- Sclerosis,
- Stroke,
- Total knee Replacement.
- Young Homeless Refugees

Although caring for older people is in frequent demand, it is not looked at any of the articles in this Research Topic.

Likewise, a range of research methodologies have been applied in the articles. Most findings are based on cross sectional research of carers' responses to a number of questionnaires in each study, some of which have already been verified, as well as constructing new questionnaires and verifying their validity and reliability.

The foci of the questionnaires range from looking at dimensions of care burden, such as anticipatory grief, anxiety, care rewards, care benefit, depression, parental perspective on the quality of life of children with a disability, Posttraumatic stress, and the carers' quality of life.

Some articles provide a systematic review of the existing literature, indicating through narrative synthesis potential improvements in caring which would be of help to the family member in need of caring and to the caregivers too (San Juan et al.).

A few of the articles aim at evaluating training in enhancing the skills that carers have and their resilience (e.g., Sharbafshaaer et al.), and introducing new forms of the support they can offer to their ill relatives, such as motivational interviewing by carers for adolescents undergoing first episode of psychosis (Kline et al.).

Sample size varies among the different studies, from 12 (in a study of telemedicine during the COVID-19 pandemic by Sharbafshaaer et al.), to 395 (Zhou et al.) for the articles which focus on measuring responses either to questionnaires or to training. Samples range in most such studies between 200 and 300+ (13, 73, 233, 243, 254, 363), enabling a reasonable degree of representation.

Statistical expertise was demonstrated in particular in one article where a network analysis of family caregivers' needs when their family member experiences cancer is outlined (Yang W. F. Z.).

Only one article follows the qualitative autoethnographic approach in providing a narrative of three episodes of being cared for. It is also the only article to be written by a service user about her experience of being cared for by her mother, and the support she has received from a health provider in enabling her to discuss discomforting experiences of care in the context of her gradual journey toward recovery and increased independence (Fox). This article highlights the value of adhering to the Triangle of Care, consisting of the person, the parent-carer, and the health professional.

Individual interviews were applied in the study of homeless youth refugees in Canada (1).

The context of the specific illness dictates to a great extent the type and degree of care required from informal caregivers, but the different articles highlight the high level of care entailed, and the high impact caregiving has on different aspects of the lives of the carers.

Most articles did not focus on policy issues and on entitlement of carers to specific support, be it financial, shared care with other family members, payment to caregivers for their input, reduction in working hours, and enabling respite care for the caregivers. However, the authors of one of the systematic reviews (San Juan et al.) indicated the lack of sufficient studies of low income countries. This study recommends that future research will entail policy and practice aspects, as well as socio-political aspects of caregiving in the different countries. This article also compares the perspective of service users vs. the perspective of the caregivers on the issue of recovery from severe mental illness.

The articles in this Research Topic provide a unique contribution to understanding the issues caregivers face, and how they make sense of their role in a range of illnesses and social contexts.

## Author contributions

SR have reviewed several of the articles and acted as the first associate editor of this Research Topic of Frontiers Psychiatry.
